# Ultrasonographic detection of enthesitis and its relation to clinical manifestations among Egyptian systemic sclerosis patients

**DOI:** 10.1186/s43166-021-00099-6

**Published:** 2022-02-08

**Authors:** Sally A. El-Leithy, Rasha M. Hammoda, Amal I. Othman, Nermin H. El-Gharbawy

**Affiliations:** 1 Physical Medicine, Rheumatology, and Rehabilitation Department, Faculty of Medicine, Ain Shams University, 36 Rabaa Estsmary Buidings, Nozha Street, Cairo, Egypt; 2 Internal Medicine and Rheumatology Department, Faculty of Medicine, Ain Shams University, Cairo, Egypt; 3 Radiodiagnosis Department, Faculty of Medicine, Ain Shams University, Cairo, Egypt

**Keywords:** Systemic sclerosis, Enthesitis, Systemic manifestations, Nailfold video capillaroscopy

## Abstract

**Background:**

Systemic sclerosis is a complex multi-systemic autoimmune disease with a wide range of its clinical manifestations; many systemic sclerosis (SSc) patients develop musculoskeletal manifestations during their course of illness. The aim of the study is to assess the prevalence of sonographically detected entheseal alterations in a case-control study of systemic sclerosis patients and to evaluate the relationship between the presence of these alterations and the clinical systemic manifestations. Patients and controls were evaluated using B mode and power Doppler ultrasonography to detect presence of enthesitis and were scored using Madrid Sonography Enthesitis Index (MASEI).

**Results:**

In SSc patients, the MASEI score was significantly higher than in control (*P* < 0.0001). Enthesitis was more prevalent among SSc patients compared to healthy controls, SSc patients with enthesitis had significantly more prevalence of diffuse subtypes (*P* < 0.001).SSc patients had significantly more prevalence of interstitial pulmonary fibrosis (IPF) (*P* < 0.001), digital ulcers (*P* < 0.001), pulmonary hypertension (*P* < 0.001), and arthralgia and arthritis (*P* < 0.001). Regarding nailfold capillaroscopy pattern, late pattern was significantly more prevalent among patients with enthesitis (*P* = 0.008). Age, ESR level, and modified Rodnan skin score were predictors for MASEI score.

**Conclusions:**

Ultrasound features of enthesopathy were frequently presented in systemic sclerosis patients. The enthesopathy was correlated with inflammation and disease complications.

## Background

Systemic sclerosis (SSc) is a chronic autoimmune multi-systemic connective tissue disease in which pathological landmarks constitute of autoimmunity, vasculopathy, and fibrosis of skin and internal organs [1]. Scleroderma is 4–5 times more common in females; average age at time of disease onset is 50 years [1]. The clinical recognizable disease is classified on the basis of extent of skin involvement into subsets with diffuse cutaneous involvement (dcSSc) and limited cutaneous involvement (lcSSc) [2]. Many patients with scleroderma develop musculoskeletal manifestations during the course of their illness in the form of arthralgia, arthritis, myalgia, stiffness of the joints, and flexion contracture [3]. Organ-specific and non-organ-specific impairments lead to a spectrum of mild to severe limitations in physical, work, and social activities [2].

The European League against Rheumatism (EULAR) Scleroderma trial demonstrated that the degree of articular involvement is associated with systemic inflammation, disease progression, and functional disability [4].

Enthesis is the site where tendons, ligaments, aponeurosis, fascia, or joint capsule become inserted into the bone [5]. Enthesitis is a condition in which enthesis became inflamed and it is one of the crucial clinical characteristics and primary pathology of spondylo-arthropathies [5]. It is also an important musculoskeletal feature in systemic sclerosis [5].

Ultrasonography has been proven to be highly sensitive, widely available, low cost, non-invasive repeatable imaging technique which does not have ionizing radiation risk [6]. It is considered as a valuable tool in the diagnosis, monitoring of joint status, and a powerful adjuvant to clinical examination as it can detect subclinical abnormalities of soft tissues, tendons, and ligaments [6]. It can be used in diagnosis and prognostic stratification of enthesitis [7].

In the literature, the prevalence of enthesitis, its clinical importance, and its correlation with different scleroderma disease parameters have not sufficiently investigated; therefore, the aim of this study was to assess the presence of enthesitis using ultrasound technique and to demonstrate the relationship between the presence of enthesitis and clinical systemic manifestations in SSc patients.

## Methods

A case-control study of forty SSc patients was recruited from the Rheumatology Out-Patients Clinic of Ain Shams University Hospitals. Enrolled patients fulfilled 2013 American College of Rheumatology/European League against Rheumatism for SSc [8]. Patients excluded from the study are those with spondyloarthropathy or any other associated autoimmune diseases, history of corticosteroid injection at any entheses sites or intra-articular corticosteroid injection into joints adjacent to entheses sites, history of malignancy, trauma, active infections, diabetes mellitus, hyperuricemia, and history of severe uncontrolled medical illness.

Twenty healthy controls were recruited from hospital staff or visitors who did not have any musculoskeletal diseases. Informed consent was obtained from all individuals in the study. Research ethics in Ain Shams University approved the study.

All patients were subjected to careful history taking, general and musculoskeletal examination, screening of clinical manifestations, and organs involvement. Disease duration was defined as time interval since onset of Raynaud phenomenon or skin symptoms. The severity of skin affection was evaluated using modified Rodnan skin score [9].

All participants were subjected to complete blood picture (Coulter counter), erythrocyte sedimentation rate (ESR) (Westergren method), fasting blood sugar, and 2-h post-prandial blood sugar (glucose oxidase method) and serum uric acid (uricase method).

Transthoracic echocardiography was done for those patients with scleroderma using two-dimensional, M-mode, color flow, and spectral Doppler techniques [10]. Mean PAP > or equal 25 with pulmonary capillary wedge pressure (PCWP) < or equal 15 mmHg confirmed having PAH according to2015 ESC/ERS guidelines for the diagnosis and treatment of pulmonary hypertension [11].

*Nailfold video capillaroscopy* (*NVC*) was performed for all patients using (Optilia Digital Capillaroscopy System, Sweden). Patients were asked not to take caffeinated drinks for at least 4 h before NVC and to remain at least 15 min in temperature 22-23 degree. Nail fold of 2nd, 3rd, 4th, and 5th were examined by using video capillaroscopy equipped with ×200 magnification contact lens. Four consecutive fields extending over 1 mm in the middle of nail fold were studied per finger [12]. The following parameters were considered: the shape and diameter of the capillaries, the capillary length, the mean capillary density which is normally 9-11/ linear mm, and the avascular area when (inter capillary distance ≥ 500 μm) [13]. The following definitions were used for the qualitative assessment of NVC patterns. The early pattern: the combination of few enlarged/giant capillaries, few capillary micro hemorrhages, a relatively well-preserved capillary distribution, and no evident loss of capillaries. The active pattern which includes frequent giant capillaries, frequent capillary micro hemorrhages, moderate loss of capillaries, mild disorganization of the capillary architecture, and absent or mild ramified capillaries. The late pattern: in which irregular enlargement of capillaries, few or absent giant capillaries, and micro hemorrhages, severe loss of capillaries with large avascular areas, disorganization of the normal capillary array, neoangeogenisis, and ramified/bushy capillaries are present [14].

### CT technique

High-resolution computed chest tomography scan (HRCT) was done for scleroderma patients to assess the presence of interstitial pulmonary fibrosis using multidetector CT machine (General Electric (GE), optima660, 128 slice).

### Ultrasound technique

US scanning of the entheses were performed by two rheumatologists trained in musculoskeletal and blinded to patients’ characteristics and clinical features for all participants. Ultrasound machine used was: (LOGIQ 9 pro series; GE Medical systems, USA) equipped with (7-12 MHz) linear transducer.

- ➢ The following entheses sites included in The Madrid Sonography Enthesitis Index (MASEI) score were assessed [15]:

- Six entheses sites (distal triceps tendon, distal quadriceps tendon, distal and proximal patellar ligament insertion, distal Achilles tendon, and proximal plantar aponeurosis) were scanned bilaterally in axial and longitudinal planes. In this scoring system, the total possible score on both sides (12 entheses) is 136, value > 18 is the cutoff point [15].

b) Patient position: The triceps tendon examined while the patient was seated with his arm resting on the examination table sitting in front of the examiner with the elbow flexed. Patients were placed in a supine position to assess the patellar and quadriceps entheses. The knee was placed in 70-degree flexion to assess knee abnormalities. Then, the patients were placed in a prone position with the feet over the end of the examination table for the assessment of Achilles tendon and plantar fascia enthesis.

- ➢ Normal gray-scale US, B mode, and power Doppler US appearance of enthesis:
- The normal insertional tract of a tendon shows fibrillary echotexture (fine parallel hyperechoic lines, each separated from the others by sonolucent interspaces) up to 1–2 mm from the cortical sub-entheseal bone.
- The sub-entheseal bone cortex shows a sharp hyperechoic profile (mild irregularities of the surface are frequent in normal entheses).
- In power Doppler study, color gain was set at a level just below the disappearance of color noise deep to the cortical bone, normal power Doppler of enthesis is absent or low vascularity [16].

- ➢ The following gray-scale US findings indicative of enthesitis:

Thickening and hypoechogenicity of the tendon insertion (the thickness of the enthesis was measured at the insertion of the deeper tendon margin into the bone in a longitudinal axis) bone erosions, bursitis, and enthesophyte formation.

### Scoring system

- ➢ Calcifications
- Score 0 = absent.
- Score 1 = if a small calcification or ossification was present.
- Score 2 = if clear presence of enthesophytes.
- Score3 = if large calcifications and ossifications were present.
- ➢ Bursitis
- Score = 0 if absent.
- Score = 1 if a well-circumscribed, localized anechoic, or hypoechoic area was detected at the site of a bursa.
- ➢ Bony erosion was defined as a cortical breakage with a step-down bone contour defect.
- Score = 0 if absent.
- Score = 3 if present.

- ➢ Tendon and ligament thicknesses were measured at the point of maximal thickness on the bony insertion. Planter aponeurosis thickness should be < 4.4 mm, Achilles tendon thickness should be < 5.29 mm, quadriceps tendon thickness should be < 6.1 mm, triceps tendon thickness should be < 4.3 mm, and patellar ligament thickness should be < 4 mm [15].

-B mode and power Doppler signal in entheses sites and tendons/ligaments were evaluated.

### Statistical analysis

The collected data was revised, coded, tabulated, and introduced to a PC using Statistical Package for Social Science version 20 (SPSS-V20, USA). Data was presented and suitable analysis was done according to type of data obtained for each parameter. Mean ± SD and range for numerical data, frequency, and percentage for non-numerical data. Student *T* test was used to compare between two groups in quantitative data. Linear correlation coefficient used to assess the strength of association between nonparametric variables in same group. Results were considered significant at *P* ≤ 0.05 and highly significant at *P* ≤ 0.001. Linear regression: It is used to test and estimate the dependence of a quantitative variable based on its relationship to one or more independent variables. Spearman’s rank correlation coefficient (rs) was used to assess the degree of association between two sets of variables if one or both of them was skewed.

## Results

The study included forty systemic sclerosis female patients and twenty healthy females as a control group. The age of patients group ranged from 33 to 59 years with a mean 42.44 ± 7.34 while for control group the age ranged from 30 to 55 years with a mean 40.80 ± 8.93. The disease duration ranged from 5 to 19 years with a mean 9.79 ± 4.39. ESR level in systemic sclerosis patients ranged from 21 to 55 with mean 39.22 ± 13.77 while in control group ESR level ranged from 10-19 with mean 14.89 ± 3.08. Modified Rodnan skin score ranged from 0-51 with mean 12.50 ± 3.12. Nailfold video capillaroscopy showed late pattern in 19 patients (47.5%), active pattern in 13 patients (32.5%), early pattern in 4 patients (10.0%), and normal pattern in 4 patients (10.0%). MASEI score ranged from 2-25 with mean 16.32 ± 7.27 in patient group while in control group ranged from 1-5 with mean 3.06 ± 1.52 (Table 1). According to presence or absence of enthesitis, twenty-five (62.5%) patients had enthesitis and fifteen (37.5%) patients had no enthesitis.

**Table 1:** Demographic and clinical data of systemic sclerosis patients and controls

	Patient (***n*** = 40)			Control (***n*** = 20)
	**Mean (SD)**			**Mean (SD)**
**Age**	42.44 ± 7.34			40.80 ± 8.93
**Duration of disease**	9.79 ± 4.39			-
**ESR level**	39.22 ± 13.77			14.89 ± 3.08
**MASEI score**	16.32 ± 7.27			3.06 ± 1.52
**Modified Rodnan skin score**	12.50 ± 3.12			-
		***N***	**%**	
**Nail fold capillaroscopy**	*Normal*	4	10.0%	-
	*Early*	4	10.0%	
	*Active*	13	32.5%	
	*Late*	19	47.5%	

Values are expressed as mean ± SD and number (%), *ESR* erythrocyte sedimentation score, *MASEI* Madrid Scoring Enthesitis Index

On comparing both groups regarding demographic, laboratory data, and disease characteristics, patients with enthesitis had significantly more diffuse subtype (*P* < 0.001) and significant more prevalence of CT detected interstitial pulmonary fibrosis (IPF) (*P* < 0.001), pulmonary hypertension (PH) (*P* < 0.001), digital ulcer (*P* < 0.001), and presence of arthritis and arthralgia (*P* < 0.001) and significantly more prevalence of late scleroderma pattern (*P* = 0.008) (Table 2).

**Table 2:** Comparison between scleroderma patients with and without enthesitis detected by ultrasound regarding demographic, clinical, and laboratory data

Scleroderma patients, ***n*** = 40				
Parameter	All (***n*** = 40)	Pt. with enthesitis (***n*** = 25)	Pt. without enthesitis (***n*** = 15)	Sig.
**Age (years)**	42.44 ± 7.34	41.32 ± 6.32	43.56 ± 8.36	0.312
**Sex (female)**	20 (100.0%)	13 (100%)	7 (100%)	1.000
**Disease duration (years)**	9.79 ± 4.39	10.20 ± 4.28	9.38 ± 4.49	0.535
**MASEI**	16.32 ± 7.27	18.56 ± 4.08	10.20 ± 4.20	< 0.001**
**Clinical manifestations**				
*LcSSc*	21 (52.5%)	7 (28%)	14 (93.3%)	< 0.001**
*DcSSc*	19 (47.5%)	18 (72%)	1 (6.7%)	< 0.001**
*Raynaud’s*	25 (62.5%)	15 (60.0%)	10 (66.7%)	0.676
*IPF*	27 (67.5%)	22 (88.0%)	5 (33.3%)	< 0.001**
*Digital ulcer*	21 (52.5%)	19 (76.0%)	2 (13.3%)	< 0.001**
*Pulmonary hypertension*	15 (37.5%)	11 (44.0%)	4 (26.7%)	< 0.001**
*Telangiectasia*	8 (20.0%)	6 (24.0%)	2 (13.3%)	0.419
*Esophageal dysmobility*	33 (82.5%)	21 (84.0%)	12 (80.0%)	0.750
*Arthritis/arthralgia*	24 (60.0%)	20 (80.0%)	4 (26.7%)	< 0.001**
**Laboratory investigations**				
Hemoglobin (g/dl)	11.30 ± 2.32	10.81 ± 2.22	11.78 ± 2.40	0.167
WBC (10^9^/l)	7.57 ± 2.73	7.24 ± 2.55	7.89 ± 2.91	0.429
Platelets (10^9^/l)	268.72 ± 61.23	257.14 ± 57.22	280.29 ± 65.23	0.212
ESR (mm/1st h)	39.22 ± 13.77	57.83 ± 15.71	20.60 ± 11.83	< 0.001**
**Nailfold capillary pattern**				
Early pattern	4 (10.0%)	1 (4%)	3 (20%)	0.107
Active	13 (32.5%)	8 (32%)	5 (33.3%)	0.933
Late	19 (47.5%)	16 (64%)	3 (20%)	0.008*
Normal pattern	4 (10.0%)	0 (0.0%)	4 (26.7%)	0.007*

Using: =, independent sample *t* test; ≠, Mann-Whitney test; ▲, Fisher’s exact; ■, Chi-square test; *P* > 0.05 NS; *P ≤ 0.05 S, **P ≤ 0.001 HS; *SSc*, systemic sclerosis; *MASEI*, Madrid Scoring Enthesitis Index; *LcSSc*, limited type; *DcSSc*, diffuse type; *IPF*, interstitial pulmonary fibrosis; *ESR*, erythrocyte sedimentation rate; *WBC*, white blood cells

Comparing both groups regarding nail fold video capillaroscopy results, patients with enthesitis had significantly lower capillary density (*P* = 0.015), significantly larger capillary width (*P* < 0.001) and significant presence of dilated and mega-capillaries (*P* = 0.002) (Table 3).

**Table 3:** Comparison between scleroderma patients with and without enthesitis detected by ultrasound according to various nailfold capillaroscopic findings

	SSc patients (***n*** = 40)			
Parameter	All (***n*** = 40)	With enthesitis (***n*** = 25)	Without (***n*** = 15)	***P***
Capillary density	7.75 ± 2.43	6.73 ± 2.33	8.77 ± 2.63	0.015*
Capillary length	190.64 ± 36.21	185.44 ± 35.19	193.70 ± 37.23	0.486
Capillary width	41.21 ± 7.85	51.51 ± 9.79	36.11 ± 6.83	< 0.001**
Capillary hemorrhage	15 (37.5%)	11 (44.0%)	4 (26.7%)	0.280
***Capillary shape***				
Normal shape	8 (20.0%)	1 (4.0%)	7 (46.7%)	0.002*
Dilated mega-capillaries	28 (70.0%)	22 (88.0%)	6 (40.0%)	0.002*
Tortuous capillaries	4 (10.0%)	2 (8.0%)	2 (13.3%)	0.593
Sub-capillary venous plexus	27 (67.5%)	18 (72.0%)	9 (60.0%)	0.439

Using: =, independent sample *t* test; ≠, Mann-Whitney test; ▲, Fisher’s exact; *P* > 0.05 NS; *P ≤ 0.05 S, **P ≤ 0.001 HS; *SSc*, systemic sclerosis

On displaying the best fitting multiple linear regression model for MASEI score, we found that ESR level, modified Rodnan skin score, dcSSc, pulmonary hypertension, arthralgia and arthritis, late pattern, and capillary width of nailfold capillary pattern were dependent predictors for MASEI score (Table 4).

**Table 4:** Best fitting multiple linear regression models for the MASEI score

Parameters	***Β***	***SE***	***t*** test	***P*** value
ESR level	6.523	0.113	7.123	0.005
Modified Rodnan skin score	7.632	0.133	7.764	0.004*
Nail capillaroscopy	0.355	0.072	0.970	0.052
**Clinical manifestations**				
*DcSsc*	2.543	0.044	2.379	0.016*
*LcSsc*	0.295	0.663	0.454	0.602
*Raynaud’s*	0.139	0.029	1.571	0.154
*IPF*	0.885	1.991	1.362	0.163
*Digital ulcer*	0.345	0.776	0.530	0.500
*Pulmonary hypertension*	2.975	0.052	2.784	0.013*
*Telangiectasia*	0.162	0.033	1.838	0.127
*Esophageal dysmobility*	1.036	2.329	1.593	0.136
*Arthritis/arthralgia*	8.929	0.155	6.314	0.003*
**Laboratory investigations**				
Hemoglobin (g/dl)	1.212	2.724	1.865	0.113
*WBC (10*^*9*^*/l)*	0.486	0.099	3.477	0.035*
Platelets (10^9^/l)	0.415	0.085	1.655	0.063
**Nailfold capillary pattern**				
Early pattern	0.404	0.908	0.621	0.415
Active	0.190	0.039	1.131	0.106
*Late*	10.447	0.182	3.654	0.003*
Normal pattern	0.472	1.062	0.726	0.344
*Capillary density*	0.190	0.039	1.131	0.106
Capillary length	0.221	0.045	1.496	0.088
*Capillary width*	3.481	0.060	3.257	0.011*
Capillary hemorrhage	0.553	1.242	0.851	0.286
***Capillary shape***				
Normal shape	0.259	0.053	1.925	0.073
Dilated- mega-capillaries	0.647	1.455	0.995	0.238
Tortuous capillaries	0.303	0.062	1.405	0.061
Sub-capillary venous plexus	0.757	1.701	1.164	0.197

*R* = 0.623; Model ANOVA: *F* = 7.514, < 0.05, using linear regression analysis, *P* > 0.05 NS; *P ≤ 0.05 S; *LcSSc* limited type, *DcSSc* diffuse type, *IPF* interstitial pulmonary fibrosis, *ESR* erythrocyte sedimentation rate, *WBC* white blood cells

The distribution of ultrasound findings detected compatible with entheses alterations revealed that the most frequent pathology was increased thickness of the tendon insertion (80%) of cases, calcifications (40%), enthesophytes (12%), erosions (12%), and presence of power Doppler signal abnormalities (8%) (Table 5).

**Table 5:** The distribution of ultrasound findings detected compatible with enthesis alterations

Pathology	Number of patients	Percentage %
Increased tendon thickness	20	80%
Calcifications	10	40%
Bony Erosions	3	12%
Positive power Doppler signal	2	8%

**Fig. 1:**
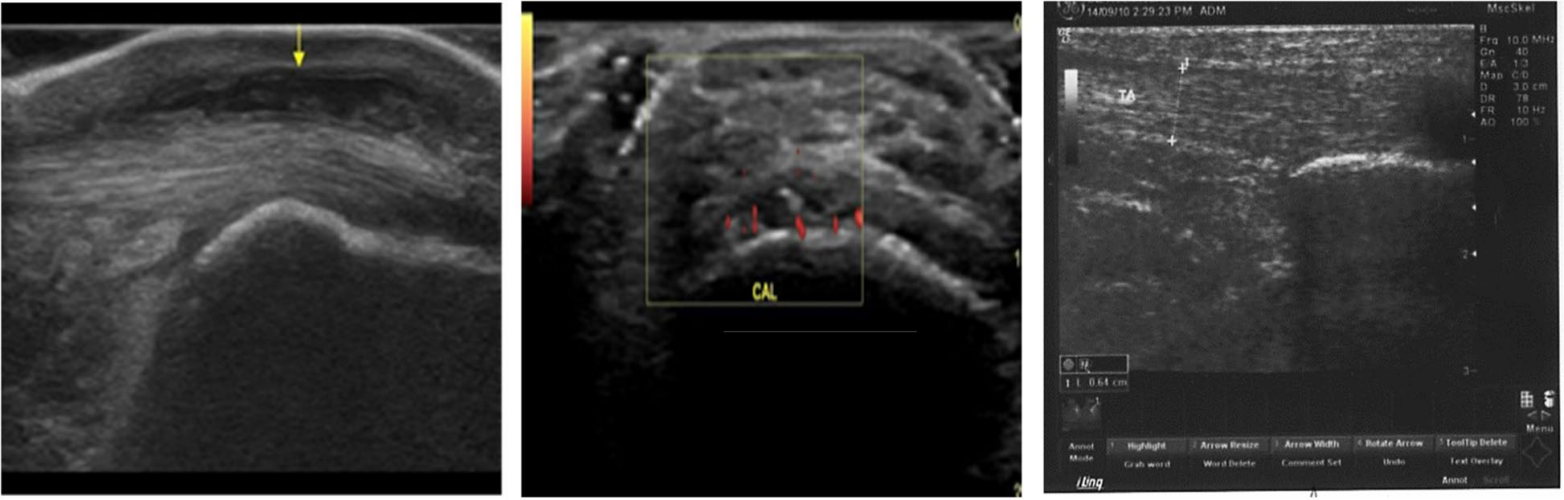
**A** Longitudinal B-mode superficial ultrasound of the dorsal ankle showing retro calcaneal bursitis seen as hypoechoic thick walled fluid filled bursa at the retro calcaneal space. **B** Longitudinal color Doppler superficial ultrasound of the planter aspect of the foot showing thickened planter fascia with increased vascularity denoting planter fasciitis, cal (calcaneus(. **C** Longitudinal B-mode superficial ultrasound at the level of the dorsal ankle showing thickened tendo-Achilles tendon (between calibers 6.4 mm)

**Fig. 2:**
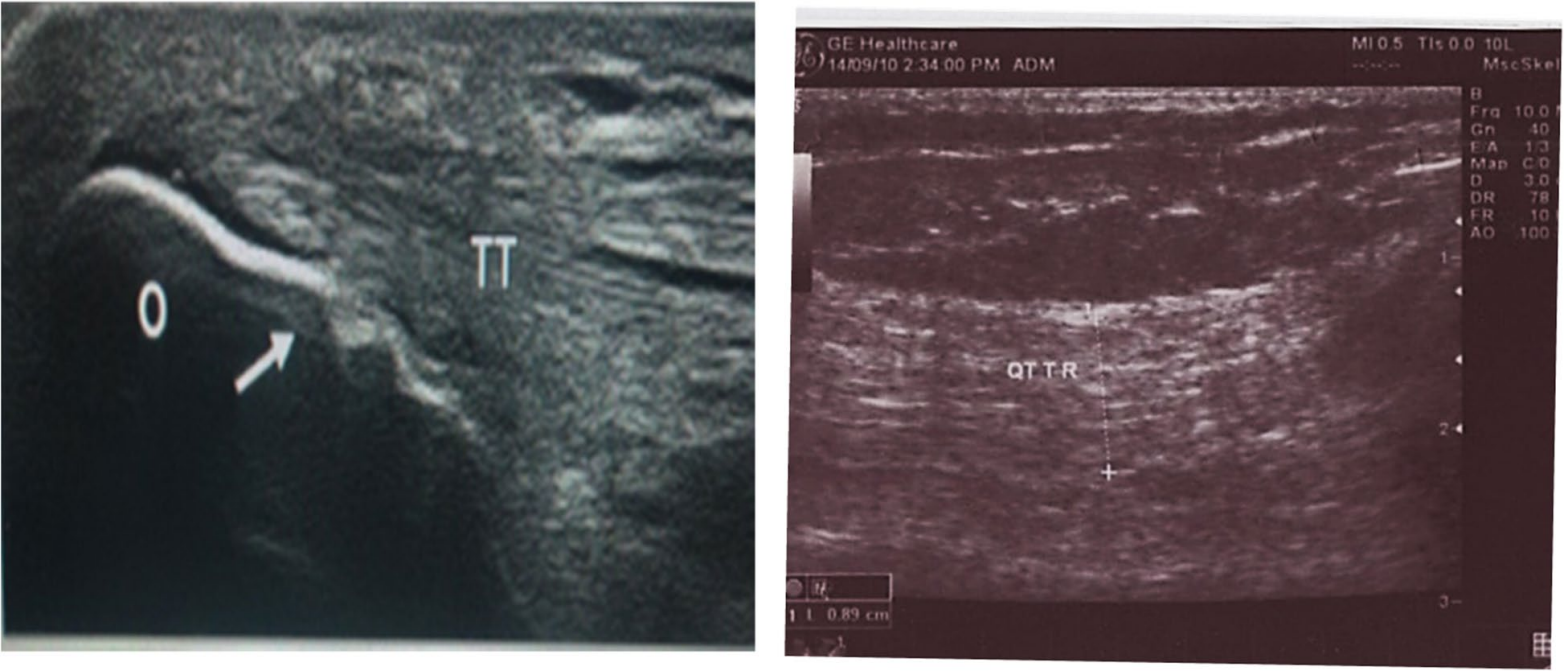
**A** Longitudinal B-mode superficial ultrasound of the elbow at the site of insertion of triceps tendon (TT) at the olecranon (O) showing irregularities and erosion of the olecranon (white arrow). **B** Longitudinal B-mode superficial ultrasound at the level of the knee joint showing thickened right quadriceps tendon (QT) (8.9 mm)

Regarding the anatomical distribution of the ultrasound detected sites; the most frequent affected entheses were the quadriceps and patellar tendons (30% each), plantar aponeurosis (37%), and distal triceps tendon (3%) (Figs. 1 and 2).

## Discussion

Systemic sclerosis is a chronic complex autoimmune multi-systemic disease which characterized by the presence of fibro proliferative condition in the microvasculature, several symptoms reported in this illness which include Raynaud phenomenon, gastroesophageal reflux disease, skin thickening, and joint manifestations which are reported by 24-79%in patients with scleroderma [17].

Arthralgia was reported as the most prevalent joint manifestation; however, inflammatory arthritis and enthesitis were considered infrequent [7].

In our study, gray scale and power Doppler US were used to investigate six entheses sites using MASEI. The six entheses sites examined were the same as investigated by Kilic et al. [6].

On comparing scleroderma patients with healthy control, we reported that MASEI score were significantly higher among scleroderma patients. These results were similar to those demonstrated by Kilic et al. [6], as they concluded that scleroderma patients had significantly higher MASEI score than healthy control subjects.

The current study concluded that scleroderma patients frequently presented with ultrasonographic features of enthesopathy compared to healthy controls.

Also, these results agreed with those of Trenziet al [1]., as their study also demonstrated that SSc patients commonly present US B-mode features of enthesopathy at entheseal insertions. Besides, their analyses also revealed that US B-mode alterations and power Doppler US signals of entheses were significantly more frequent in SSc than in controls [1].

Moreover, in Kilic et al. study, they found that except for plantar aponeurosis, MASEI scores and all tendon and ligament thickness were higher in patients with SSc compared with those in the control [6].

Among our scleroderma patients, 62.5% were diagnosed to have enthesitis which was relatively similar to Schanz et al. [18] who detected enthesitis in 56% of his patients, also Kilic et al. [19] reported that 44.2% of his scleroderma patients had enthesitis.

Both magnetic resonance imaging (MRI) and US can be used to demonstrate enthesitis, but MRI has some disadvantages, that is, it is expensive, has low accessibility, is inconvenient to some of the patients, lacks sensitivity and specificity for peripheral enthesitis and is unable to image multiple entheses sites simultaneously. It has been shown that US is a valid and reliable assessment tool for the evaluation of enthesitis and it is superior in showing structural change, calcification, and inflammatory activity in the tendons and ligaments. Therefore, US can be used as the preferred method for the detection and follow-up of enthesitis or enthesopathy [6].

The distribution of ultrasound findings detected compatible with entheses alterations revealed that the most frequent pathology was increased thickness of the tendon insertion (80%) of cases, calcifications (40%), enthesophytes (12%), erosions (12%), and presence of power Doppler signal abnormalities (8%).These findings were in agreement with Kilic et al. [19], where they found that the most frequent pathology was thick tendon and ligaments followed by erosions and then entheses calcifications and power Doppler signal abnormalities [19].

Regarding the anatomical distribution of the ultrasound detected sites, the most frequent affected entheses were the quadriceps and patellar tendons (30% each), plantar aponeurosis (37%), and distal triceps tendon (3%).

These results are in agreement with Kilic [6], where the most frequent sites where quadriceps and patellar tendons but in their study the least affected site was the planter aponeurosis yet in our study, the lowest frequent affected site was the triceps tendon.

Diffuse skin disease is more likely to be associated with higher risk of internal organ damage and musculoskeletal involvement [20]. On comparing patients with enthesitis and those without according to extent of skin fibrosis patients with enthesitis were more of the diffuse subtype; this was similar to the study of Stoenoiu et al. [20] and Avouc et al. [4]. As they reported that tendon involvement occurred more frequently among diffuse scleroderma patients than limited subtype.

On the other hand, Terenzi et al. [1] found no association between enthesitis and disease subtypes. Furthermore, Kilic et al. [19] reported that there was no difference in MASEI score among patients with limited and diffuse subtypes.

Disease duration plays an important role in different scleroderma systemic involvement [4]; however, we reported no difference regarding disease duration among patients with enthesitis and those without which was in disagreement with the findings of Fawzy study [17], who concluded that incidence of synovitis and tendon affection were significantly higher among those patients with disease duration more than 3 years. Moreover, the present study revealed that scleroderma patients with enthesitis significantly had more prevalence of interstitial pulmonary fibrosis, digital ulcer, pulmonary hypertension, and arthralgia which was in concordance with Kilic et al. [19] study who reported that there was positive correlation between MASEI score and dyspnea grade. Also, Avouc et al. [21] concluded that synovitis joint contracture and tendon involvement were associated with more severe disease and with systemic inflammation.

Regarding the laboratory data, the patients in our study with enthesitis had significantly higher ESR which appeared in line with Schanz et al. [18], who reported that every patient with synovitis and enthesitis had elevated CRP which reflects ongoing inflammatory state.

In systemic sclerosis, the pulmonary system is frequently involved and causes a significant increase in morbidity and mortality. It is estimated that 80% of patients with SSc have some degree of pulmonary affection which makes pulmonary system the second most commonly affected visceral system after esophagus [22].Ground-glass opacities, lung architectural distortion which reflects lung fibrosis is often prominent and lobar volume loss is seen in cases of more advanced fibrosis [23].

Nailfold capillaroscopic examination is a highly sensitive, simple, easy, and safe technique which plays an important role in the diagnosis of pattern scleroderma; on studying the frequency of NVC scleroderma (early—active—late), patients with enthesitis had significantly higher prevalence of late pattern which was in agreement with Avouc et al. [21].

On correlating MASEI score with age, disease duration, modified Rodnan skin score, we observed positive correlations between MASEI score and all these parameters which was in accordance with Kilic et al. [19], who detected positive correlations between MASEI score with age and modified Rodnan skin score.

On displaying the best fitting multiple linear regression models for MASEI score, we concluded that ESR level, modified Rodnan skin score, dcSSc, pulmonary hypertension, arthralgia/arthritis, late pattern, and capillary width of nailfold capillary pattern were dependent predictors for MASEI score.

This agreed with Kilic et al. [19], who found positive correlation between MASEI score and age, dyspnea grade and Rodnan score; however, he attributed increase age in those scleroderma patients with enthesitis due to degenerative changes that developed with age.

## Conclusions

- Enthesitis was detected among scleroderma patients using musculoskeletal ultrasound (MSUS) and its presence was associated with more severe multi-systemic disease manifestations.
- MSUS is a sensitive tool for the detection of enthesitis in SSc patients.

## Limitations of the study

- Limited sample size and lack of follow-up study due to poor compliance of the patients and the fear of COVID-19 infection.
- Lack of fund limited the possibility of adding an important investigation tool as pulmonary function tests.

## Recommendations

- Increase sample size and duration of the study
- Using other radiological modality as MRI and comparing it with ultrasound to identify which is more sensitive in detecting subclinical enthesitis among scleroderma patients

## Data Availability

The datasets used and/or analyzed during the current study are available from the corresponding author on reasonable request.

## Abbreviations

SSc: Systemic sclerosis
dcSSc: Diffuse cutaneous involvement
lcSSc: Limited cutaneous involvement
MASEI: Madrid Sonography Enthesitis Index
EULAR: The European League against Rheumatism
ESR: Erythrocyte sedimentation rate
RVSP: Right ventricular systolic pressure
RV: Right ventricle
PAP: Pulmonary arterial pressure
PCWP: Pulmonary capillary wedge pressure
NVC: Nailfold video capillaroscopy
HRCT: High-resolution computed chest tomography scan
MDCT: Multidetector CT computerized tomography
IPF: Interstitial pulmonary fibrosis
PH: Pulmonary hypertension
